# Supplementary material for: A curated dataset for data-driven turbulence modelling
Source: Sci Data. 2021 Sep 30;8:255. doi: 10.1038/s41597-021-01034-2 (PMC8484471; doi:10.1038/s41597-021-01034-2)
Supplement: Supplementary file 1 — Supplementary Figures [file 41597_2021_1034_MOESM1_ESM.pdf]

A curated dataset for data-driven turbulence modelling

Supplementary information

List of Figures

1 Profiles of U for three meshes of varying density for the $\alpha = 0.5$ periodic hills case. 2
2 Profiles of V for three meshes of varying density for the $\alpha = 0.5$ periodic hills case. 2
3 Profiles of U for three meshes of varying density for the $Re = 3,500$ square duct case. 3
4 Profiles of U for three meshes of varying density for the $h = 42$ mm parametric bump case. 3
5 Profiles of V for three meshes of varying density for the $h = 42$ mm parametric bump case. 4
6 Profiles of U for three meshes of varying density for the $Re = 20,580$ converging-diverging channel case. 4
7 Profiles of V for three meshes of varying density for the $Re = 20,580$ converging-diverging channel case. 4
8 Profiles of U for three meshes of varying density for the curved backward-facing step case. 5
9 Profiles of V for three meshes of varying density for the curved backward-facing step case. 5

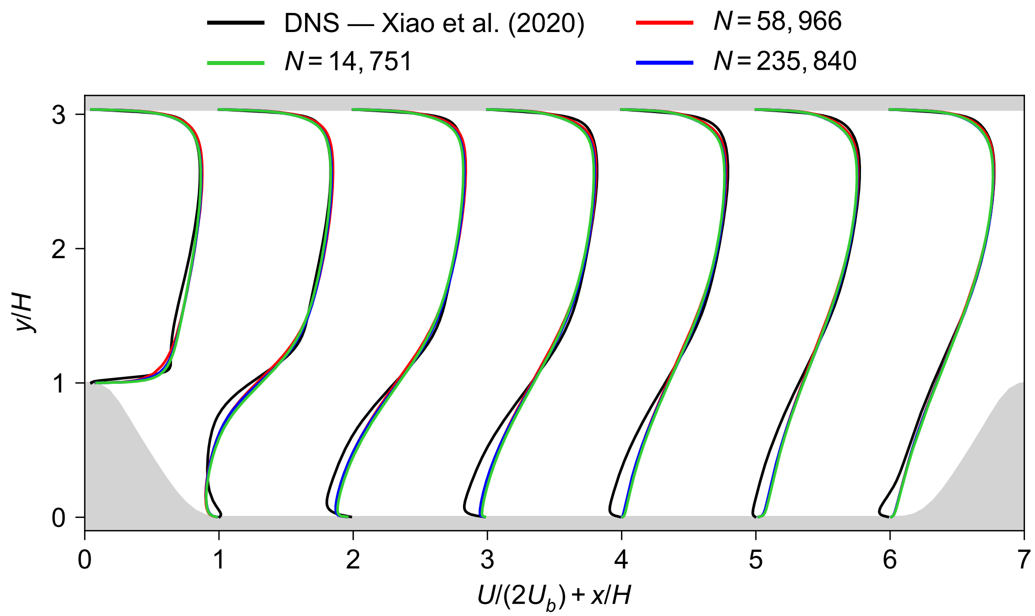

Supplementary Figure 1. Profiles of U for three meshes of varying density for the $\alpha = 0.5$ periodic hills case.

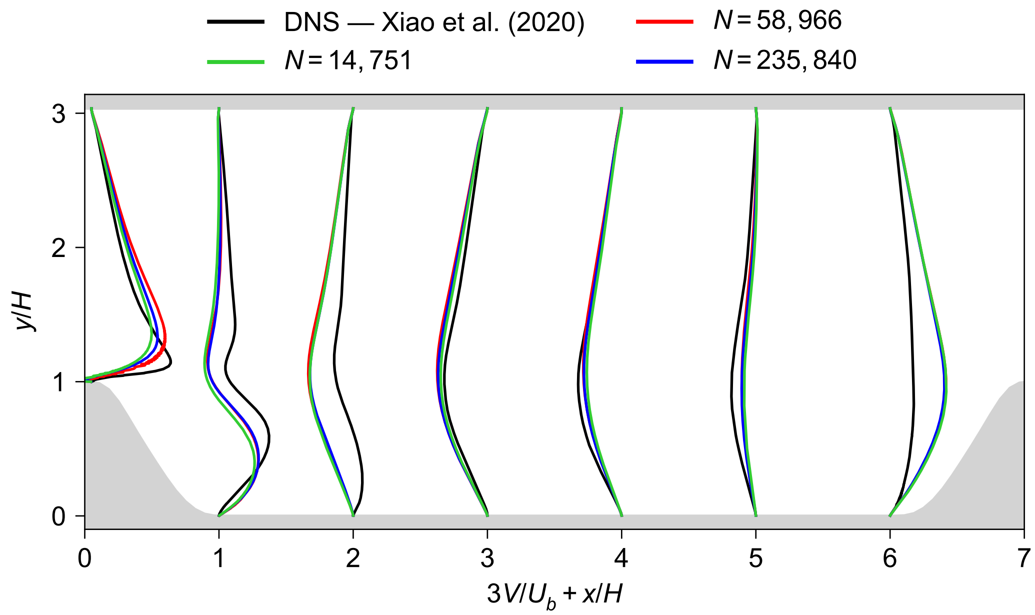

Supplementary Figure 2. Profiles of V for three meshes of varying density for the $\alpha = 0.5$ periodic hills case.

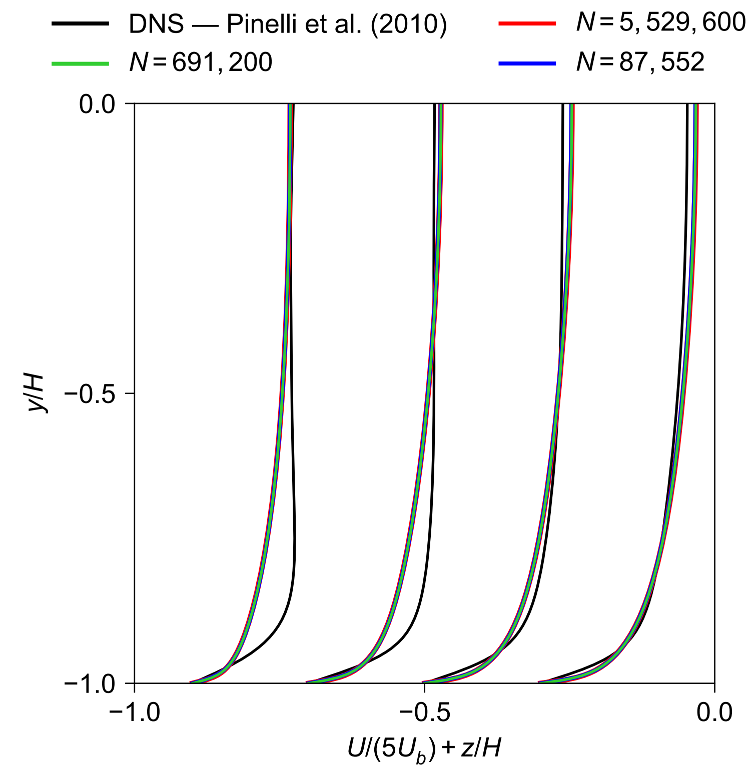

Supplementary Figure 3. Profiles of U for three meshes of varying density for the $Re = 3,500$ square duct case.

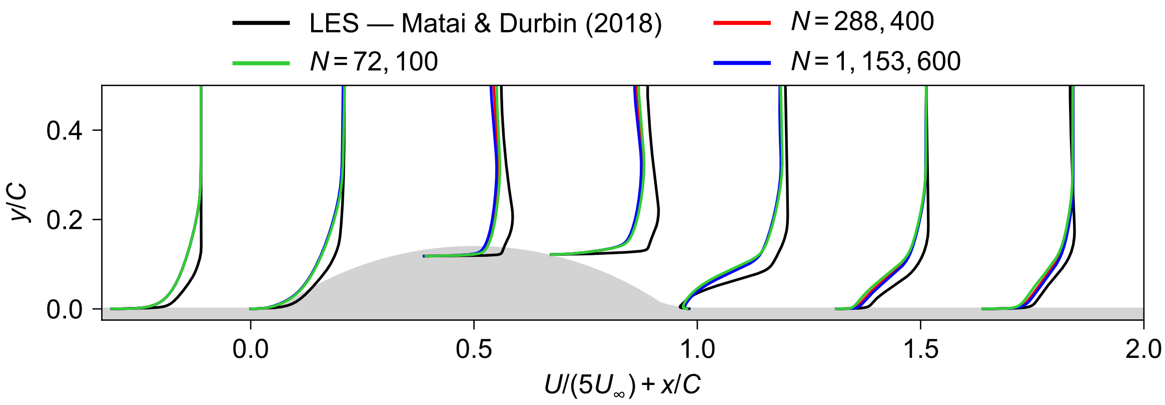

Supplementary Figure 4. Profiles of U for three meshes of varying density for the $h = 42$ mm parametric bump case.

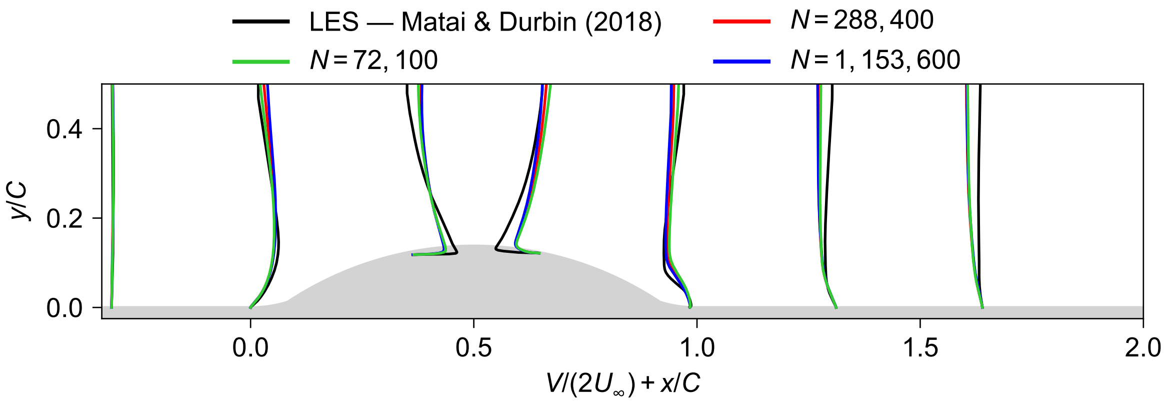

Supplementary Figure 5. Profiles of V for three meshes of varying density for the $h = 42$ mm parametric bump case.

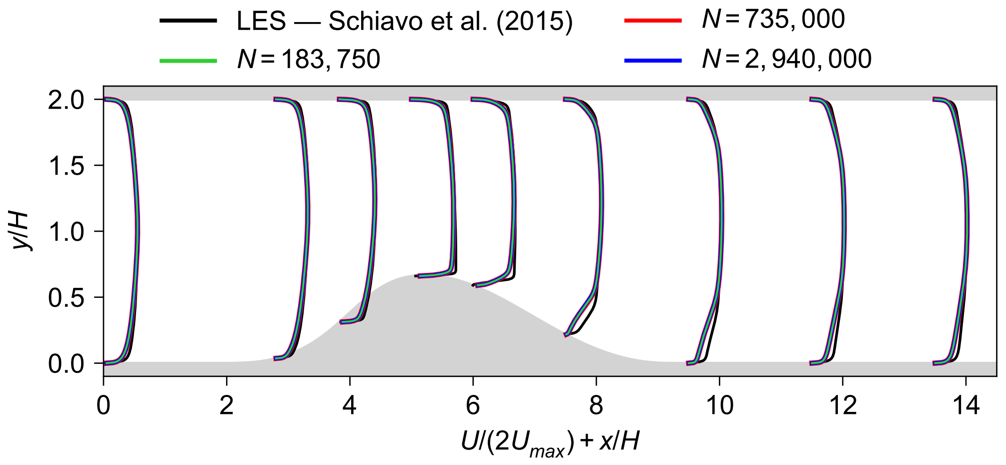

Supplementary Figure 6. Profiles of U for three meshes of varying density for the $Re = 20,580$ converging-diverging channel case.

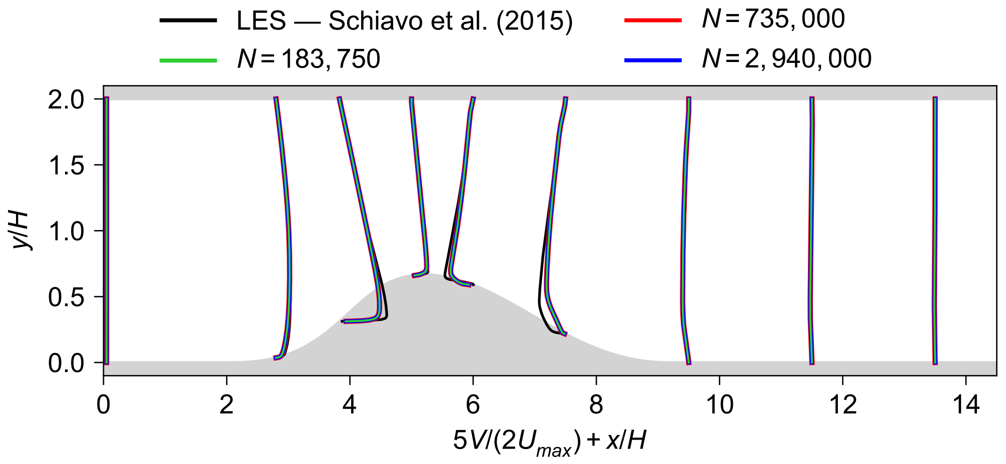

Supplementary Figure 7. Profiles of V for three meshes of varying density for the $Re = 20,580$ converging-diverging channel case.

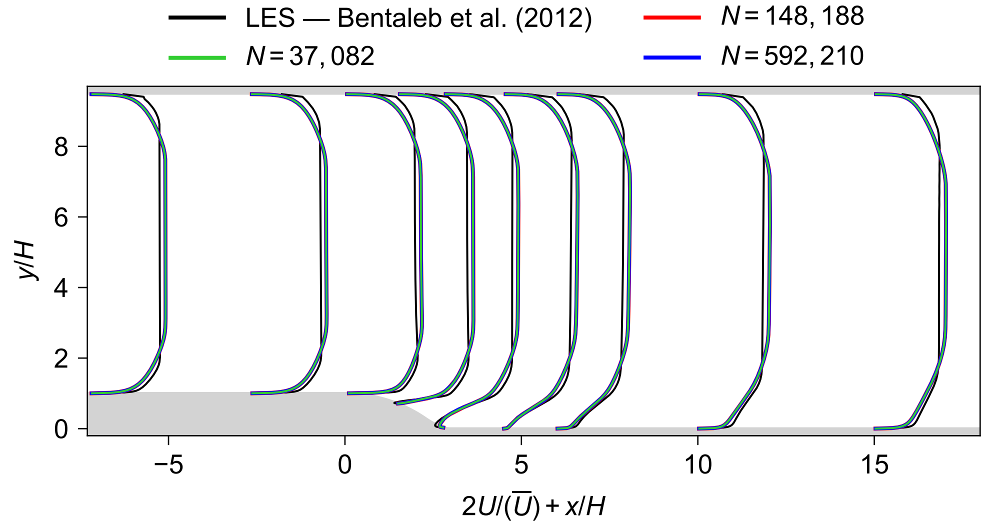

Supplementary Figure 8. Profiles of U for three meshes of varying density for the curved backward-facing step case.

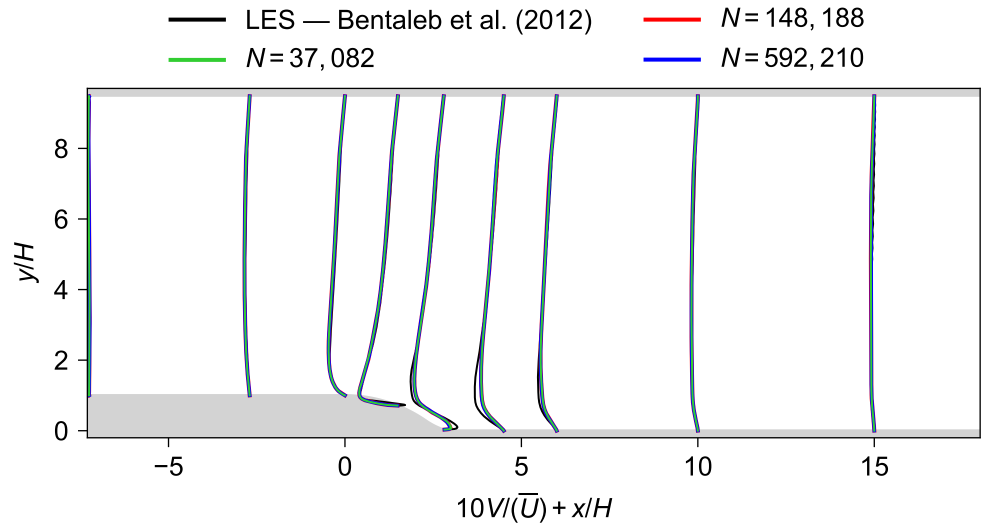

Supplementary Figure 9. Profiles of V for three meshes of varying density for the curved backward-facing step case.
